# The pull of soccer and the push of Xhosa boys in an HIV and drug abuse intervention in the Western Cape, South Africa

**DOI:** 10.1080/17290376.2018.1541024

**Published:** 2018-11-14

**Authors:** Melissa Medich, Deborah Mindry, Mark Tomlinson, Mary Jane Rotheram-Borus, Jason Bantjes, Dallas Swendeman

**Affiliations:** a Department of Family Medicine, University of California Los Angeles, Los Angeles, CA, USA; b Center of Expertise on Women’s Health, Gender and Empowerment, University of California Global Health Institute, San Francisco, CA, USA; c Department of Psychology, Stellenbosch University, Stellenbosch, South Africa; d Global Center for Children and Families, University of California Los Angeles, Los Angeles, CA, USA; e Department of Psychiatry & Biobehavioral Sciences, David Geffen School of Medicine, University of California Los Angeles, Los Angeles, CA, USA

**Keywords:** HIV/AIDS, South Africa, masculinity, soccer, drugs, gender, violence

## Abstract

There is growing interest in engaging men and boys in health and development programmes targeting the intersection of HIV risk, substance abuse, and violence. Understanding the conceptualisations of masculinities or masculine identities that shape both behaviours and opportunities for intervention is central to advancing the global agenda to engage men in health and development interventions. This paper examines an intervention using soccer and job training to engage and deliver activities for HIV prevention, substance abuse, and gender-based violence in a South African township. A literature review provides theoretical, historical and social context for the intersection of gender, masculinity, soccer, violence, and sexual relationships. Qualitative data from in-depth interviews and focus groups is analysed using theoretical and contextual frames to elucidate the negotiation of shifting, contradictory, and conflicting masculine roles. Results highlight how changing risky, normative behaviours among young men is a negotiated process entailing men's relationships with women and with other men.

## Introduction

South Africa has the highest number of seropositive persons of any nation with an estimated 7 million people living with HIV and an estimated 2.4 million HIV-infected men (UNAIDS, 2017; UNICEF, 2018; WHO, 2014). Young men living in townships in South Africa are at high risk for substance use, unemployment, and HIV, yet they have been mostly excluded from intervention programmes due to their alcohol and drug use, gambling, and dysfunctional male group dynamics (Fleming et al., 2015; Rotheram-Borus et al., 2016; Yunus, 2003). Most evidence-based interventions are neither highly attractive to men nor consistent with masculine coping styles (Fleming et al., 2015; Messner, 1997; Taylor, 2007). Behaviour change in public health interventions for all populations, particularly young men, has been notoriously difficult to initiate and sustain (Rotheram-Borus et al., 2016). Furthermore, the theory, counseling-based models, and clinical or organisational settings that dominate evidence-based HIV prevention interventions have not previously been driven by theories of masculinity or models of constructive masculine engagement (Michielsen et al., 2010; Steinberg, 2008).

In recent years global health programming with men has increasingly shifted from being gender-neutral to gender-sensitive or gender-transformative (Barker et al., 2010; Dworkin, Fleming, & Colvin, 2015). Global health programmes have increasingly targeted social determinants of health in the form of economic and social factors (e.g. race, class, etc.) that influence the stability of communities, groups, and individuals to attain good health (Dworkin et al., 2015; Marmot, 2008). In South Africa, men's sexual behaviours have been identified as the prime driver of the HIV epidemic and have generated great interest in male sexuality (Gevers, Mathews, Cupp, Russell, & Jewkes, 2013; Mathews, Jewkes, & Abrahams, 2011; Stern, Clarfelt, & Buikema, 2015). Nevertheless there has been limited understanding of how gender relations drive men's risk behaviours and their health (Blankenship, Friedman, Dworkin, & Mantell, 2006; Dworkin, Colvin, Hatcher, & Peacock, 2012; Dworkin, Fullilove, & Peacock, 2009).

Over the last decade there has been increasing interest in and attempts to engage South African men in HIV prevention and care through interventions focused on changing gender norms (Balfour et al., 2013; Dworkin et al., 2012, 2015; Gevers et al., 2013; Jewkes, Flood, & Lang, 2015; Mathews et al., 2011; Pettifor et al., 2010; Rotheram-Borus et al., 2016; Shefer, Kruger, & Schepers, 2015; Stern & Buikema, 2013). Theory-based gender-transformative interventions focused on changing men's relations with women in South Africa and in Sweden have mobilised the concept of hegemonic masculinity, which explains how and why some men are positioned to maintain dominant social roles over women, and over other men in subordinate social positions (Connell & Messerschmidt, 2005; Flood, 2014; Jewkes, Flood, et al., 2015; Jewkes, Morrell, et al., 2015; Kato-Wallace, Barker, Eads, & Levtov, 2014; Leddy, Chakravarty, Dladla, de Bruyn, & Darbes, 2015). These studies enrich our understanding of how gender relations drive men's health in terms of male/female gender dynamics. However, there is a gap in understanding how gender norms and power dynamics play out in male/male social relationships, particularly in the context of public health interventions addressing the intersection of masculinities and interpersonal violence, substance use, and HIV/AIDS (Jewkes, Flood, et al., 2015).

This paper aims to help fill this gap in the literature through a qualitative analysis of data from a pilot study of an HIV prevention intervention, the ‘Champions League’ with young Xhosa men living in townships in the Western Cape Province of South Africa. The ‘Champions League’ uses a culturally compelling strategy of soccer (football) to engage young men in activities to promote prevention of drug abuse, HIV, and gender-based violence, such as behavioural modelling, drug/alcohol and HIV testing, and educational sessions integrated throughout practices and games, and vocational training opportunities offered to participants demonstrating consistency of prosocial and responsible behaviours in practices and games, such as showing up on time and sober, good sportsmanship, non-violence, and respect for coaches and players (see Rotheram-Borus et al., 2016, 2018 for detailed descriptions of the intervention).

Culturally compelling interventions rely on a thorough understanding of local social and ecological landscapes that shape behaviours and the links between the intention to change, actual behaviour change, and the subsequent health impact (Panter-Brick, Clarke, Lomas, Pinder, & Lindsay, 2006). In South Africa soccer provides a strong sense of national identity (Sewpaul, 2009) and is strongly associated with masculine identity. Programmes that utilise soccer as a tool for social change are steadily emerging in townships and rural areas (Coalter, 2009; Levermore, 2008, 2010; McGhee, 2012) and a number of organisations have used the popularity of football (soccer) in Africa to engage youth in HIV prevention strategies based on national, regional, and ethnic identities (Balfour et al., 2013; Clark, Friedrich, Ndlovu, Neilands, & McFarland, 2006; Langer, 2015; Ley, Lembethe, & Chiware, 2013; Maro, Roberts, & Sorenson, 2009). For example, in the Cape Flats of the Western Cape, there are We Are Not Statistics (WANS) and Otherlands Football Academy (OFA). The OFA participants self-identify as ‘Afrikkans-speaking coloureds’, whereas WANS participants self-identify as Xhosa (McGhee, 2012) similar to Champions League participants.

### Gender, theory, and health

Gender is a social determinant of health like race, class, and working conditions (Marmot, 2008). Courtenay’s (2000) foundational article on men's health noted the inability of health-related research to explain men's risk-taking that perpetuates the widespread, yet false, cultural assumption that such behaviours are biologically natural or inherent. Global health scholars have now empirically shown that men who experience gender role conflict – when they fear that they cannot live up to masculine gender norms – have worse health outcomes (Bertozzi & Gutiérrez, 2013; Dworkin et al., 2015; Pettifor et al., 2010; Pleck, 1995). Recent advances in theory and applied research have started to explain how social constructions of masculinity and male gender norms are important contributors to men's disproportionate enactments of violence, sexual, and other risk behaviours, as well as how power dynamics manifest in their relationships with intimate female partners (Dworkin et al., 2015; Dworkin, Treves-Kagan, & Lippman, 2013; Fleming et al., 2015; Jewkes, Morrell, et al., 2015; Leddy et al., 2015; Mathews et al., 2011; Mmeje et al., 2016; Shefer et al., 2015; Stern et al., 2015).

‘Gender role’ theory explains how individuals are socialised to act according to the expectations associated with one's biological sex (Connell, 1987, 2005). Global health scholars drew on the concept of gender roles in health programmes, including the more recent critiques and expansions of the theory that extend beyond individualised, fixed notions of gender, and address the significance of power relations (Connell, 1987; Connell & Messerschmidt, 2005; Messner, 1997). A broader understanding of masculinity is envisioned as an ideology or set of beliefs manifested as dynamic patterns of social relationships enacted by individuals and patterned into social institutions such as sports, the military, gangs, etc. In other words, gender is not something that one ‘is’ but rather is something one ‘does’ in a patterned set of social interactions within institutions (Courtenay, 2000). Gender, as a verb, as it is performed, is relational and built into patterns of social practices and dynamic social structures that are at once contested, fluid, and locally and regionally determined (Dworkin et al., 2015; Ortner, 1996; Strathern, 1988).

Much of the theoretical work on men and masculinities revolves around the concept of hegemonic masculinity (Coles, 2009), which explains that men's power over women, and other men, is perpetuated by particular groups of men inhabiting positions of power and wealth that reproduce and legitimate social relationships that generate their dominance (Carrigan, Connell, & Lee, 1985). Hegemonic masculine norms value male ‘toughness’, virility, and dominance over women, with men subscribing to these norms being more likely to engage in HIV risk behaviours (Blankenship et al., 2006; Campbell & Macphail, 2002; Leddy et al., 2015). Research in diverse communities in South Africa has documented how dominant, hegemonic masculinities are shaped by physicality and violent performances of masculinities (Balfour et al., 2013; Black et al., 2014; Ganle, 2015; Gevers et al., 2013; Leddy et al., 2015; Morrell, Jewkes, & Lindegger, 2012; Stern & Buikema, 2013), which are key in framing heterosexual practices that temper against safe and equitable sexual practices (Kaufman, Shefer, Crawford, Simbayi, & Kalichman, 2008; Shefer et al., 2015). Hegemonic masculinity has been criticised for emphasising structural factors and a resulting lack of attention to understanding how power is organised in terms of complicity and resistance at the individual level (Coles, 2009).

Bourdieu's theory of practice enhances the concept of hegemonic masculinity by crossing the structure-agency (individual) divide in explaining how objective structures and subjective experience shape how individuals’ navigate and negotiate practices in everyday life (Bourdieu, 1984), typically without consciousness or strategic intention (Calhoun, LiPuma, & Postone, 1993). In the current research, the soccer field is a key physical and relational domain of men's social lives that invokes performances of masculinities that point to the struggle and contestation for social positions that exist relationally between men, like coaches and players in the Champions League in the Western Cape.

### Xhosa men and masculinity

In South Africa, as elsewhere, the determination to become ‘a man’ is a powerful feature of masculinity. In *Changing Men in Southern Africa* (Morrell, 2001) Epstein and Johnson maintain that boys and men are not entirely free to choose images that please them, and thus are restricted by *fields* of social life. Historically, indigenous black South Africans responding to changes in political and social power of colonialism and apartheid formed township defense committees and are characterised by Xaba (Xaba, 2001) as ‘struggle masculinity’ and ‘post-struggle masculinity’. During apartheid, struggle masculinity valued ‘liberation now and education later’. Defense committee members had the perception that Xhosa elders tolerated and accommodated apartheid, thus creating generational tension in the townships. Status as a Young Lion or Liberator (examples of defense committees) brought social respect and psychologically satiating accolades, including being coveted by women (Xaba, 2001). Nonetheless, particular configurations of masculinities forged in one historical moment can become obsolete and dangerous in others (Xaba, 2001), as witnessed in South Africa. Young males who became exiled hero soldiers in the 1980s were desperate in the 1990s when they returned to South Africa because they lacked documents and professional skills. Thus, the former exiles turned to violent crime and displays of deadly bravado such as committing gang rapes and revenge killings. However, while there are no doubts that the conditions in apartheid South Africa affected constructions of masculinity, viewing such conditions in a deterministic way is precarious and merits more critical analyses.

Traditional Xhosa culture envisions men as economic providers, respectful of elders, and protectors of family and community; however, this contradicts historical understandings of pre-colonial societies. Historically, Southern African men were not the sole economic providers of their households; they were their figurative heads. The processes of European colonialism escalated or exaggerated gender tensions (Isike, 2012). In contemporary townships, ‘successful’ or ‘dominant’ masculinity constitutes sexual, aggressive relationships with girls and struggles for position and status among male peers (Wood & Jewkes, 2001). Broader attitudes about different types of violence in townships are tolerated, even expected behaviours. The perception is that violence, particularly mild ‘disciplinary’ forms, were acceptable given male entitlements to women and the importance of men asserting power in their sexual relationships (Wood & Jewkes, 2001).

### Circumcision as a defining feature of manhood in Xhosa culture

Circumcised Xhosa men are expected to take on greater social responsibility in their communities, negotiating family disputes, carefully weighing decisions, and cooperating with elders (Vincent, 2008). Through the dichotomous lens of traditional rites of passage and circumcision, boys have many sexual partners while men have one while considering marriage. In reality, many who profess respect for initiation teachings seem to quickly lapse into pre-circumcision behaviours and expectations (Vincent, 2008). Xhosa community and traditional leaders agree that the role of circumcision schools has eroded to the extent that circumcision is now regarded as a gateway to sex rather than as a marking point from which responsible sexual behaviour begins (Vincent, 2008). Xhosa men, like men in many contexts globally, have clear incentives for two competing discourses. Combining the providing husband/father with the playboy, intersects with common contemporary socio-sexual contexts that are saturated with themes of violence, familial breakdown, resource scarcity, and inter-generational conflict. Initiates generally accept physical punishment as part of the process of transition to manhood. Notably, the education young men receive in the bush at traditional circumcision schools – that a defining characteristic of masculinity is respect for others and non-violence – conflicts with the norms of violence prevailing in township life (Wood & Jewkes, 2001). Regardless, ritual rites of passage like circumcision have remained in Xhosa culture and are strongly correlated with separation of son from mother, integration of man into the community, and preparation for marriage, adult sexuality, and warfare (Block, 1986; Gluckman, 1949; Turner, 1962; Vincent, 2008).

### Gangs

A detailed discussion of warfare and masculinity is beyond the scope of this paper, but gang violence merits brief mention as it is a component of contemporary township life discussed by the youth in the Champions League. Gang history in South Africa is recorded back to the early twentieth century, but by the late 1930s and 40s the ‘*tsotsi*’ identity emerged among urbanised working-class youth (Glaser, 1992).

Male *tsotsis* were structurally dominant with their gender serving as the one sphere where they could assert themselves as ‘naturally’ privileged. Gangs merged with political activists after the 1976 anti-apartheid uprisings and subsequently separated into dangerous, non-overtly political street gangs in the 1980s and 1990s. At that time township gangs were infamous for violent sexual assaults against women such as ‘jackrolling’ (gang rape) (McGhee, 2012). Currently, gangs are linked to drug trafficking networks centred in areas like the Cape Flats, such as *Gugulethu* (our pride) and *Khayelitsha* (our new home) townships where the Champions League took place. Gangs are ubiquitous and commit violent crimes like rape, murder, robbery, etc. to the point where they are written about on a local Cape Flats webpage (http://capeflats.org.za/modules/home/gangs.php). They have considerable political power in townships by controlling the movement of people through and around established gang territories.

### The historical *pull* of football

The first documented game of football played in South Africa took place between British soldiers and employees of the colonial administration in August 1862 in the Cape Peninsula (McGhee, 2012). In the Natal Colony, European missionaries used soccer to lure Zulu men into attending mission schools (Alegi, 2010). After South Africa officially became the Union of South Africa (1910), many black African men started migrating to urban areas and soccer's popularity grew. It was very inexpensive and relatively easy to learn to play. It was a leisure activity for men attempting to establish lives with unfamiliar people in unfamiliar locations. British settlers used it to form bonds with other immigrants to the Cape Peninsula (Carton, 2000). Alegi (Alegi, 2004) recounts that soccer was ‘transformed into a sphere of action where expressions of African modernity could be forged, tested, and negotiated’ during the early twentieth century.

Black Africans formed their own soccer clubs in Durban beginning in 1916. However, during the segregation period (1926–1940) a succession of governmental Acts restricted the movement and autonomy of black people. Black Africans from several ethnicities and separated by social class distinctions (e.g. mission-educated elites, migrant and non-migrant workers) started socialising through local soccer club affiliations. Soccer functioned as a vehicle for changing urban, black masculine identities by helping club members affirm their self-worth and contributing to a dominant, working-class athletic masculinity (Alegi, 2004). This athletic masculinity included an element of violence. Violent challenges to referee calls (another symbolic authority figure) intensified and transformed the football grounds into sites of physical conflicts, and riots (Alegi, 2004). During apartheid (1948–1994), soccer was a political tool against racist oppression. Participating in soccer became a form of rebellion against apartheid policies. Soccer proved difficult for government officials to police because it is easy to play on most surfaces with little equipment. A series of boycotts by international sport institutions including FIFA and the International Olympic Committee occurred in tandem with economic sanctions that left South Africa's economy crippled. Interestingly, in the post-apartheid era, soccer transformed from a tool for political rebellion to a tool for political reconciliation. For example, Nelson Mandela's presidential inauguration included a soccer match between the South African national team and Zambia. Then, South Africa was awarded the honour of hosting the 2010 FIFA World Cup, the first held on the continent of Africa. The political, economic, and symbolic significance of the 2010 World Cup caused much debate in the country rife with social and economic problems (Alegi & Bolsmann, 2010). Nonetheless, soccer remains a central domain for the expression of masculinity, particularly among black South Africans.

### The ‘Champions League’ soccer intervention

The Champions League intervention was implemented through Grassroots Soccer, an international community-based organisation, in April 2012 and ran for six months. A detailed description of the intervention and its feasibility and preliminary impacts from a pilot study are published elsewhere (Rotheram-Borus et al., 2016), as is the protocol and updated intervention description currently being tested in a cluster-randomised trial (Rotheram-Borus et al., 2018). Briefly, coaches were selected by Grassroots from the broader township rather than from target neighbourhoods to ensure confidentiality. They were trained to reinforce HIV risk reduction norms and healthy daily living framed by enhancing personal and team performance. Coaches’ responsibilities included creating role-plays and discussing: (1) the consequences and effects of long-term alcohol and drug use on the body, family, community, and society; (2) how to interact effectively with health care providers, partners, and family members about one's health, HIV, HIV testing, diabetes, TB, and drug abuse; (3) how to create enjoyable daily routines and healthy social networks, including with women, emphasising respectful and caring manners; (4) the benefits of exercise; and (5) young men's relationships with women and gender-based violence. The intervention had a points-based system, which rewarded participants during practices and games for being punctual, drug-free, and cooperative during practices, games, and life-skills sessions, and remaining for the entire practice/game times. Young male participants were incentivised via contingency management to take drug and alcohol tests on a random basis every week during the six months intervention period. All of these intervention activities were integrated throughout practices and games. A longer-term incentive was eligibility for vocational training opportunities and equipment that required adhering to the incentivised code of conduct. The combination of soccer and job training aims to promote social and personal behaviours that emphasise responsible and respectful relationships.

## Methods

### Procedures and participants

This study received IRB approval from both UCLA and Stellenbosch University. Qualitative data were collected via in-depth interviews and focus groups of intervention participants – players, family members, and coaches. Interviews were conducted before focus groups to ensure individual perspectives were not contaminated by views expressed in focus group discussions. An anthropologist researcher assisted in the qualitative data design, development of the interview guides, reviewed transcripts, and led data coding and analysis. Trained research assistants, who were bilingual in Xhosa and English, conducted the interviews and focus groups.

Semi-structured interview guides were developed by the research team to give specific attention to substance abuse, HIV, gender relations, and finances. The interview and focus group guides, and method of administering the interview minimised biases by asking open-ended questions before more direct probes. In-depth interviews ascertained what participants and their families understood about the programme and their perception of the impact of the programme on young men's behaviours and attitudes. Focus groups with players and coaches gathered data on their experiences, what they learned, and the changes that they experienced or observed as a consequence of the intervention. Another publication in process examines the qualitative data from this study in reference to intervention functions, processes, and participant experiences. This paper presents results related to themes on masculinities arising from the analysis.

Thirty-five key informant interviews were conducted with five coaches, 15 players and 15 family members. Three focus groups were conducted with 45 players (about 15 per group) and with five coaches in a separate focus group. Table 1 summarises methods and samples.

**Table 1.** Ethnographic methods.

Data collection method	Specific data elicited	Sample description
In-depth interviews	Participants’ understanding of the program; perceptions of the impact of the programme on young men's behaviour and attitudes about substance use, HIV testing, finances, and gender-based violence	35 key informants: 5 coaches 15 players 15 family members
Focus group discussions	Gather data on participants’ experiences in the programme; what they learned about substance use, HIV, gender relations and gender-based violence; the changes participants experienced or observed as a consequence of the intervention	4 focus group discussions: 3 with players (*n* = 15) 1 with coaches (*n* = 5)

### Data analysis

Data were coded by a research assistant using Atlas.ti version 7.1.3 (GmbH Berlin) using an inductive, grounded theory approach to identify themes and subthemes that emerged as well as gender theory to guide coding. Multiple research assistants were used to code data, inter-reliability was tested, and sufficient consistency was determined. Revisions to coded data were based on a review process with the research team. Coding keys were established to describe general statements about male–male relationships. The gender theory of hegemonic masculinities and Bourdieu's theory of practice (described above) served as the framework for analysis, which offers insightful lenses for understanding male gender dynamics enacted in the pilot intervention study and in the men's daily lives. Analyses identified the following primary themes: Delineations as men versus boys, the pull of Xhosa family roles, friendship bonds, hegemonic masculinity on the soccer field (player and coach interactions), Circumcision as ‘man’ identifier, coaches as positive and negative role models, gangs and Champions League, and gender-based violence. Each theme is illustrated below with quotes from participants.

## Results

### Delineations as men versus boys

A primary theme that runs throughout the data and recurs in more specific examples is a broad concept of masculine identity split between self-identifications and perceptions of others as men versus boys. The following quote illustrates the overarching theme of boys versus men and the negotiated masculinities that occurred amidst the intervention activities, which was a significant source of conflict and difficulty in implementing the intervention:

in our Xhosa culture there is always a line between men and boys, and even before that there was a lot disrespect between men and boys which led to a lot fights between players as they also belong to different gangster groups. (Coach 1)

The quote summarises how men enact masculinity individually during their everyday life, relationally with and among each other, within the limits posed by domains of their everyday life. Champions League pilot study participants asserted their self-identity as Xhosa while dichotomising status between men and boys, referencing the historical tensions between them, and how Xhosa culture and family dynamics intersecting with proximal contextual factors of gangs, substance use, and gender-based violence shape role expectations and expressions of masculine identities.

### The pull of Xhosa family roles

Xhosa family dynamics are centred at the core of the Champions League programme. The desire of Xhosa players to obtain vocational training to support their families speaks to the social dynamics and their attempt to fill the roles of a ‘father’ and an elder male, as it is valued and performed in traditional Xhosa society.

… what I realized is that some of the players needed guidance, some a father figure and some could recognize me since I play professional football and that paved the way for me … I played a father figure role especially to the Manchester United (team) and also a role of being an elder brother to other teams because they came to me seeking advice and I also interacted with them in a such a way that I learnt something as some will come forward to alert me that so and so is high on drugs. (Coach 1)

… what I also discovered in the Champions League is that elders can learn from the younger ones and vice versa. What I liked also was the fact that we had faith and believed in the players because most of those of participated in the league were drug addicted and their families had given up on them, so by giving them the platform to express themselves, lending an ear made them felt like human being maybe their families had given up on them … To me it was not about money or anything but being passionate about football, and in my community I am a role model and so I would like to groom the younger generation to be exactly what I am in life. (Coach 5)

### Friendship bonds

Coaches and players indicated the positive effects of the programme on developing friendships:

I experienced working with strangers, people that I believed were criminals, but as we are together at Champions League we developed friendship … I did not know what to expect in the beginning as I did not know what kind of people I will be working with, their backgrounds at the same time I was excited so I had mixed feelings but in the end we developed friendship and personally I learned a lot and this has equipped me with presentation skills and also with life skills. (Coach 1)

We had a very good relationship, this program brought us together as a community, we got the opportunity to know each other, but before we use to pass each other on the street without greeting as we were strangers to each other … I did not have friends because I was always indoors and at home I was advised against friends. (Player 15)

We had a very good relationship but before the Champions we never gathered together as we were from the same community nor did we visit each other but after the League whenever there is something in the community we invite each other and visit each other … I even made friends with coaches … Out of the Champions League we were taught that we have now what we exactly we want in life and to think out of the box and also get out of our comfort zones in order to be successful in life because I have realized that there were many opportunities in the Champions League. (Player 4)

An important aim of the Champions League programme was to divert boys away from gangs and substance abuse. Negative connotations of gangsters as violent, delinquent men have evolved into popular discourse (Isike, 2012). Strong emphasis on discipline resulted in some players quitting the programme; nonetheless, those who stayed seemed to benefit from the strictness and rules in forming peer-mentors.

### Hegemonic masculinity on the soccer field

Tensions between the coaches and players were strongly evident in the interviews and focus groups:

What I experienced was a big challenge for me … it was the first time for me to work in a different environment, working with adults who challenge you on what you tell me and who think their opinion is right, they argue their views and you also learn something and get answers from their own opinions and responses. They challenge you both physically and verbally as you play together on the field they tackle and push you on the field during when you play the ball, to them the fact that you are their coach does not matter … They think they know football better than you as the coach the only thing you know is life skills, and they can be harsh towards you, scold you on the field, and after the training session when you go to the Life skills lessons you have to take charge, call them into order and take back your position as the coach. They compared their soccer skills against mine as being better in the field and also wanted to do that in the Life Skills classes … And they even challenged and asked the coach they suspect was younger than the players. (Coach 2)

It was a good experience but in the beginning it was difficult to deal with players. Some were of the same age as me, some were older than me than me and some were younger than me. Some were not paying attention and we were from the same area but as the time went by we began to understand each other and we got along. (Coach 5)

Coaches expressed their support for each other, the challenges that they faced as role models, and their strategies for negotiating respect from players who were sometimes older, or perceived to be older.

What I liked is the way we supported each other as coaches, on occasions when it happened that one of us was not available we use to stand in for each other. (C FGD)

What I also liked with coaches we used to have our own meetings where we took decisions without consulting with players and decide on what we were going to do the next day but at later stage we engaged them in our meetings to make them feel that they are part of the program and to give them the platform to have their inputs and their views and not decide for them. (C FGD)

We learned to understand each other as coaches, we learned to work together and cooperate and developed skills and matured as coaches, as we struggled before to communicate as coaches in the beginning as some were not use to shouting out players, but there were coaches also who use to make their teams run around the field for the duration of the training session, but there were also young coaches who had players who were older than him and they were challenged and disrespected by those players, there was a problem also of a short-tempered umpires and fights broke up on the field but those fights usually broke out when the teams were fighting over the loss in the game but everything will be back to normal. (C FGD)

The feedback from players and coaches speaks to the traditional Xhosa, elder male role models and some of the tensions that arise when young men of similar ages interact with each other in the context of the League and their respective roles in it. Manifestations of these tensions include physical and verbal aggression, yet coaches supported one another and found a solution to some of those tensions, e.g. including them in meetings and adjusting the way in which they communicated with players during training and in games.

### Circumcision as ‘man’ identifier

Among participants in the Champions League, status was discussed in terms of ‘traditional’ masculine identities that intersect age, economic independence, and circumcision:

Yes the fact that we were all men, we were looking at each other and judging if who is older than who? And they even challenged and asked the coach they suspect was younger than the players. That led to the topic of who was circumcised before who, that was discussed even out of the program, which wasn't the focus here, but what I liked also is that they waited for the program to come to an end and called us aside and ask us as it was something that they had been discussion for a long time amongst themselves during the course of the program. The coach in question didn't take it well also it disturbed him and he distanced himself from his players although he cared about them because he just did not like that. (Coach 2)

… there was clash on people that we were dealing with because some were men some were boys  … So it was a very sensitive practice and very scary in the beginning but after we did it I felt like we should do more of those practices … Well I was a victim because my team consists of boys only, there was no men. He had a brother who was also playing but in other team but they didn't see eye to eye, had some differences, he was younger than him but already circumcised but he was still a boy although he was the eldest and at some point I realized that they were talking to each other as I overheard the younger brother saying, ‘You are my brother even though you are not circumcised yet, the love I have for you will never change we come a long way’. (Coach 1)

Players’ opinions about coaches were distinct from those of coaches about players and, while player reactions after the six-week follow-up may be due to response bias, they were mostly positive. Interesting to note is that circumcision rather than age or status as coach seemed key in determining the hierarchy of Xhosa masculinity as well as the ability to control one's aggressive behaviour on the soccer field.

### Coaches as positive and negative role models

Intersecting with attributions of masculine statuses based on circumcision were attributions based on whether coaches were positive or negative role models, and could fulfil role expectations for a competent coach. Positive attributes related to a coach's patience, coaching skills, and sobriety. For example:

We had a good relationship as I told you that I was not good in football he was very patient with me and he does not easily get angry. (Player 11)

We had a very good relationship; he had very good coaching skills. I have never had any misunderstanding and most of the times he assigned me to responsibility of selecting the team for the game. (Player 14)

Players were particularly challenges with navigating bad role models in coaches exhibiting lack of caring of players and commitment to coaching, and intoxication at practices and games. For example,

Most of the time he never cared about his players … I am really not sure whether he tendered his resignation or he was fired but we submitted our grievances against him as players … he was not attending most practices and was seldom on the field, let me put it simply he really liked to drink … Sometimes he would come under the influence of alcohol … It did not affect us that much as I have mentioned that. I had a responsibility of being a Captain so I had to take over … he was seldom at practice. (Player 3)

The coach did very little for our team … He was good mannered and was not harsh towards us … On those occasions when he came to the field under the influence … No we did not because he is older than us so out of respect we did not have the courage to ask him but the question we use to ask ourselves as players is as drunk as he is how is he going to coach the team. So that is when [the Captain] would take responsibility actually not only those occasion he was fully in charge of the team. (Player 7)

… we had disciplined players as mentioned by this player that there were coaches who came to the field under the influence of alcohol so what actions were taken against those coaches, didn't you have a code of conduct for coaches but should a player commit an offence they were very strict towards us. So discipline must start with coaches. (P FGD1)

An encouraging consequence from the situation described above is that players were able to negotiate the problem of an unreliable coach, one who could not live up to his responsibilities as a role model figure. Players supported one another and assigned leadership roles based on a collective desire to play soccer. It affirms the assertion that masculinities are fluid, not fixed, protected and defended, constantly broken down and recreated, not inherited or acquired in a one-off way (Cornwall & Lindisfarne, 1994). It also affirms that the roles participants played in the Champions League soccer programme were the source of conflicts and conflicting messages. Here we see the struggle and contestation for social positions that exist relationally between men that Coles (Coles, 2009) writes about. Habitus, or strategy without strategic intent (Calhoun et al., 1993) is embedded within a system of hegemonic masculinity. Young men were able to resist on an individual level despite structural hierarchies remaining in place.

### Gangs and Champions League

Players and coaches openly discussed informal agreements with gangs during the Champions League programme:

Honestly speaking I will speak mostly about this one in particular because firstly I stay in Makhaya Section 19, and I use to work in Harare and between these two areas there were rivalry gang groups and we had no go area, but during the Champions League after getting to know the players and signing the contracts with the Coaches and Captain of the Clubs in the fixture that we had with the slogan ‘You will protect me and I will protect you’ in a certain way and when I give you this message you will convey it to your players also. I got the freedom back of walking freely on the streets without any fear of being harmed and also sometimes people will greet me as the Coach without even noticing them on the way and that made me feel safe and comfortable in the environment. (Coach 1)

During Champions League there was reconciliation between the rival gangs in Harare and Kuyasa, but when the program came to the end the situation was back to normal. (P FG3)

The culturally compelling *pull* of soccer in South African townships is evident in the relaxing of territorial boundaries of street gangs to permit pro-male and pro-social activities valued by larger society.

### Gender-based violence

The majority of players responded that were against abusing women but many stated that avoiding rape and assault charges was a motivating factor. They expressed the idea of respect in terms of negotiating their relationships and better communication about sex, wearing a condom, and not forcing themselves sexually. Still, some of the players contest that abuse against women was acceptable in certain instances:

‘Do you sometimes deem it necessary to physically abuse women?’ ‘Ayes’:

Yes it is necessary sometimes because they can also be abusive and you also end up losing temper and physically abuse her. (Player 2)

A little bit sometimes … Maybe a klap [hit] on the face … When she cheats on you. (Player 3)

A little bit sometimes because women can be disrespectful and if you don't, they think you are too soft or you are a gay. (Player 6)

Sometimes but you should not be too harsh towards them. There are some girls that you can see they were abused in their previous relationships and the only language they understand is abuse. (Player 9)

P: There are times you find yourselves abusing your girlfriend without meaning to do that, for example if you arrive at the tavern and men are all over your girlfriend and whenever you call her she keeps on ignoring you then you can clap her but the next morning you should apologize … . (P FDG 2)

‘Do you think it is necessary to physically abuse women sometimes?’ ‘Nays’:

No, those are steps that one goes through but as you grow up you realized that it is not a right thing to do … , I have made that decision that if we don't get along I rather leave her than abusing her. (Player 10)

No it is not necessary because women are defenseless … Rather ask her to leave if she is by in your house or if you are in the street leave her. (Player 5)

What if she attacked you first?

P: Yes women can provoke you sometimes and push you but when she does that the best thing is to walk away or rather take your training gear and get into the road and by the time you come back you will obviously be feeling better.

P: Women can provoke you sometimes and the best thing you can do is to ignore her. (P FGD 3)

## Discussion

This paper situates its analysis of qualitative data from players, coaches, and family members within the historical context of soccer in South Africa to elaborate how soccer transects the local social, economic, and political life of young men in the Western Cape. Analysing this data and the intervention processes through the lens of gender theory buttresses the push for public health intervention research to address HIV-risky, dominant male norms in a relational aspect, in order to promote sexual and reproductive health (Blankenship et al., 2006; Dworkin et al., 2009, 2012, 2015; Jana, Basu, Rotheram-Borus, & Newman, 2004). Using gender theory to analyse the relational, gender dynamics between the players and coaches is essential to understanding the acceptance of or resistance to HIV, gender-based violence, and drug abuse prevention interventions tailored for young African men as well as highlighting the importance of using culturally compelling interventions in challenging populations.

Young Xhosa men are aware of the symbolic meaning embedded in traditional adult masculine status and its direct connection with circumcision as well as the tensions of achieving that status in contemporary society. Participants expressed how men struggle to establish themselves as men and how acutely aware young men are of their struggle. Without obvious generational differences, young men utilised the traditional Xhosa symbol of adult masculine status to evoke a sense of dominance or at least to contest it. A sense of disrespect between men and boys appears to persist from the apartheid era during which more than two-thirds of children in South Africa lived with one or both parents away from home for the majority of a year (Wilson, 2006). Townships were originally designed as areas to ‘warehouse’ black African workers and emphasised keeping families apart. The most devastating effects of apartheid were the dismantling of Xhosa (also Zulu and other Bantu groups) families through forced removals via the Group Areas Act, Pass Laws, and the influence of migrant labour (Clowes, Ratele, & Shefer, 2013). Adding HIV/AIDS to the dynamic and ‘AIDS orphans’, attention shifted to household structures of dying mothers and absent fathers. Black African children (all Bantu groups together) had the greatest reported increase in fatherless homes between 1993 and 2002 which means that black African children were more likely not to reside in the same household as their fathers than to reside with them (McGhee, 2012). Some fathers were voluntarily absent from their sons’ lives while others died (AIDS, cancer, etc.), were killed in mines, or due to street violence.

Soccer is a domain where discourses take place and reinforce the paradox of hegemonic masculinity in terms of age groups differences and power. Age was a source of tension in the Champions League programme, which links to ‘traditional’ masculinities and young men's struggle to establish themselves in social settings in relationship to other men. It supports the landscape on which masculine identities are shaped by inter-group rivalry and aggressive behaviour rather than generational conflict (Mager, 1998). The theory of hegemonic masculinity considers that interpersonal violence between men is much higher than men with women (Mayer & Beyrer, 2013; Shefer et al., 2015; WHO, 2014). The men who participated in the Champions League clearly have agency but their agency is limited and dependent on socioeconomic and cultural contexts that constrain and shape their choices. Some men use masculinity linked to interpersonal violence or the threat of it as a resource to construct status when they are otherwise marginalised within broader society (Courtenay, 2000; Majors & Billison, 1992).

Milder forms of gender-based physical abuse seemed to be socially accepted in the context of highly emotional situations where men perceive women not performing their roles as women correctly; lying, cheating, physical assault on the men. South African scholars argue the history of apartheid and violent colonialism cultivated a society marked by violence (Jewkes & Morrell, 2010; Morrell et al., 2012) that produced violent masculinities (Morrell, 2001). South African masculinities carry attributes of ‘physical strength, courage, toughness, and an acceptance of hierarchical authority, but most of all, they demand that men are able to exercise control [over women and over men] (p. 5)’ (Jewkes & Morrell, 2010). Post-apartheid women now have an ability to bring legal charges against abusive men, yet also to provoke men into abusive actions because of learned gender dynamics in Xhosa society.

In describing a theory of hegemonic masculinity (Connell, 2005) and violence one must theorise the engendered subject (Moore, 1995). Individuals are subjects who take up multiple subject positions within a range of discourses and social practices; some will contradict while others conflict with each other (Moore, 1995). There is a notion of ‘investment’ in particular positions that require emotional and vested interests in terms of satisfaction, reward, or payoff. This notion is witnessed by the social interactions described in the data presented in this paper. Coaches in particular had vested interests in their positions as coaches, as did players in terms of their fellow players and the opportunity for vocational training. Crises, such as apartheid in South Africa afford opportunities to thwart historically gendered subject positions due to the pressure of expectations about self-identity/social position or the inability to receive expected satisfactions or rewards from taking up a particular gendered subject position or mode of subjectivity. For example, violence is often the outcome of an inability to control other people's sexual behaviour, or rather, other people's management of themselves as engendered individuals. In other words, the behaviour of others threatens the self-representation and social evaluations of oneself.

## Conclusions

Using soccer as a vehicle for behaviour change combined with job training is a culturally compelling development tool in South Africa (Jane Rotheram-Borus et al., 2016). It attracts a larger number of people, particularly youth (Clark et al., 2006; Coalter, 2009). The sport has also historically been associated with social transition and establishing social networks among men. Most HIV prevention interventions are demonstrated to be efficacious and then require a push into medical settings where the majority of participants are women. This study provides evidence for complex processes of negotiation in a delivery format that diverges from HIV evidence-based interventions, which tend to focus on women, as presented in the introduction. Soccer is a facilitator for friendship bonding, for discourses of masculine agendas, and for larger discussion on gender dynamics. The tension and bonding between coaches and players improved over the course of the programme, when coaches and players learned to demonstrate respect for one another. Players were able to assume leadership roles in situations where their coach would be absent or intoxicated during practices and games. Coaches were not subjected to sanctions for their antisocial behaviours and echoes concepts of hegemonic masculinities and Bourdieu's theory of practice where men negotiate, resist, and re-establish authority. Notable in interviews from both coaches and soccer players, the soccer league created a context for men from different gang affiliations to share activities and engage in camaraderie that facilitated their socialising away from the soccer pitch and in the streets. Offering young men a controlled *field* or social domain in which to enact masculine behaviours was valued by the community.

Inclusion of circumcision in future prevention projects may be valuable. In contemporary South African society, emphasis on the acquisition of circumcision and rights to marriage has become subverted into an emphasis on rights to sex and material resources, especially sharing food. Material motivations feature strongly in initiates’ accounts of the pull of circumcision, most prominently access to sex (Vincent, 2008). During communal feasts, uncircumcised boys are not allowed near where meat is prepared; they are not entitled to share but are dependent on scraps thrown to them by circumcised men. Studies by Tenge (Tenge, 2006) and Mavundla (Mavundla, Netswera, Toth, Bottoman, & Tenge, 2010) report that uninitiated boys will typically be excluded from the family's budget for new clothing, rather they receive hand-me-downs from older family members. Moreover, those who have been circumcised actively use a person's uncircumcised status to limit access to women. Vincent (Vincent, 2008) argues that while the physical features of traditional male Xhosa circumcision have essentially remained unscathed by the impacts of colonialism and apartheid, contemporary initiates do not experience the inner changes at the level of identity. Young male participants are individual agents, or actors, of a society where sexual socialisation has been disrupted by broader social ruptures in family and community life. Traditionally the initiation of young men was a communal responsibility, now it is much more of an individual endeavour. Once an overarching message of responsibility and control; masculinity has been transformed into a focus on the right of access to sex as a primary marker of manhood.

The Champions League study was conducted in an environment with a high risk for HIV infection, prevalence of gender-based violence, high numbers of unemployed young men who join gangs for lack of other activities, little or no interventions with young men, and a high desire for soccer and job training (Jane Rotheram-Borus et al., 2016). Soccer is a compelling vehicle for changing risky behaviour of young men because it articulates preexisting social priorities for facilitating and establishing male bonds and bridging those bonds into wider society.
